# Olanzapine Prevents the PCP-induced Reduction in the Neurite Outgrowth of Prefrontal Cortical Neurons via NRG1

**DOI:** 10.1038/srep19581

**Published:** 2016-01-19

**Authors:** Qingsheng Zhang, Yinghua Yu, Xu-Feng Huang

**Affiliations:** 1 Centre for Translational Neuroscience, School of Medicine, University of Wollongong, Wollongong, 2522, NSW, Australia; 2 Illawarra Health and Medical Research Institute, Wollongong, 2522, NSW, Australia; 3 Schizophrenia Research Institute, 384 Victoria Street, Darlinghurst, 2010, NSW, Australia

## Abstract

Accumulating evidence suggests that reducing neurite outgrowth and synaptic plasticity plays a critical role in the pathology of cognitive deficits in schizophrenia. The N-methyl-D-aspartate receptor antagonist phencyclidine (PCP) can induce symptoms of schizophrenia as well as reduce dendritic spine density and neurite growth. The antipsychotic drug olanzapine may improve these deficits. This study aimed to investigate: (1) if olanzapine prevents PCP-induced suppression of neurite outgrowth and synaptic protein expression; (2) if olanzapine affects the Akt-GSK3 signaling pathway; and (3) the role of neuregulin 1 (NRG1) in this process. Immunofluorescence revealed that PCP treatment for 24 hours reduces both neurite length (28.5%) and the number of neurite branches (35.6%) in primary prefrontal cortical neuron cultures. PCP reduced protein and mRNA expressions of synaptophysin (24.9% and 23.2%, respectively) and PSD95 (31.5% and 21.4%, respectively), and the protein expression of p-Akt (26.7%) and p-GSK3β (35.2%). Olanzapine co-treatment prevented these PCP-induced effects in normal neurons but not in neurons from NRG1-knockout mice. These results indicate that NRG1 mediates the preventive effects of olanzapine on the PCP-induced impairment of neurite outgrowth and synaptic protein expression. This study provides potential targets for interventions on improving the efficacy of olanzapine on preventing cognitive deficits in schizophrenia.

Schizophrenia is considered to be a neurodevelopmental disease^1^ with disturbances in synaptic connectivity being a key pathology^2^. While clinical evidence suggests that enlarged ventricles and decreased brain volume are the two most consistent structural abnormalities in schizophrenia^3^,^4^, rodent studies have found that these structural abnormalities are associated with abnormal neurite formation^5^,^6^. Moreover, shortened basilar dendrites and diminished dendrite collaterals at the prefrontal cortex (PFC) have been found in schizophrenia patients^7^,^8^,^9^. This suggests that neurite outgrowth and synaptic plasticity plays an important role in the pathology of schizophrenia, in particular relating to the cognitive deficit symptoms.

Phencyclidine (PCP), a non-competitive N-methyl-D-aspartate (NMDA) receptor antagonist, can induce schizophrenia-like behaviors in humans and rodents^10^,^11^. Applications of NMDA receptor (NMDAR) inhibitors, including PCP, MK-801, and ketamine, induce disinhibition on pyramidal neurons, leads to the excessive release of glutamate, and the full range of positive, negative and cognitive symptoms of schizophrenia in healthy humans^12^. Moreover, the effect of PCP on reducing dendritic spine density or neurite outgrowth has been reported in both *in vitro*^13^ and *in vivo*^14^ studies. However, the exact mechanism of PCP’s effect on neurite outgrowth is still unclear.

Olanzapine is a second-generation antipsychotic drug that has been reported to have some improvement effects on neurocognitive dysfunction in schizophrenia patients^15^,^16^,^17^. Along with other antipsychotics, olanzapine may also improve dendritic outgrowth and PSD95 and BDNF expression in hippocampal neuronal cultures^18^. Furthermore, the preventative effects of olanzapine on PCP-induced deficits in synaptic plasticity, pre-pulse inhibition, and cortical NMDAR expressions have also been reported in rodents^19^,^20^, suggesting a potential role of olanzapine in attenuating the cognitive dysfunctions in rodents, although the efficacies of this antipsychotic in cognitive deficits in schizophrenia is still under debate^21^. Importantly, olanzapine’s ability to reverse the PCP-induced loss of spine synapses in rat PFC has also been reported^22^.

Neuregulin 1 (NRG1) is a trophic factor that signals by stimulating its tyrosine kinase receptor, ErbB4 (a type I membrane glycoprotein that is a member of the ErbB family of tyrosine kinase receptors). It has been suggested that loss of function of NRG1 or ErbB4 causes deficits in the migration of pyramidal and GABAergic neurons, neurite outgrowth, and axon projection^23^, which is consistent with the ‘abnormal neural development’ model of schizophrenia. Interestingly, interactions between NRG1 and NMDAR have been suggested. It has been reported that NMDAR expression was reduced in NRG1 (∆TM)+/− mice, and the phosphorylation of NR2B on Tyr1472, which is key to NMDAR’s channel property, was suppressed in NRG1 (∆TM)+/− mice. These suggest that NRG1-ErbB4 may act downstream of NMDAR stimulation^23^.

We present evidence that olanzapine attenuates PCP’s inhibition effect on neurite outgrowth and synaptic plasticity, possibly through improving the PCP-induced downregulation of the NRG1 signaling pathway, and activating the downstream Akt-GSK cascade.

## Results

### PCP suppresses neurite outgrowth and synaptic protein expression

**Figure 1:**
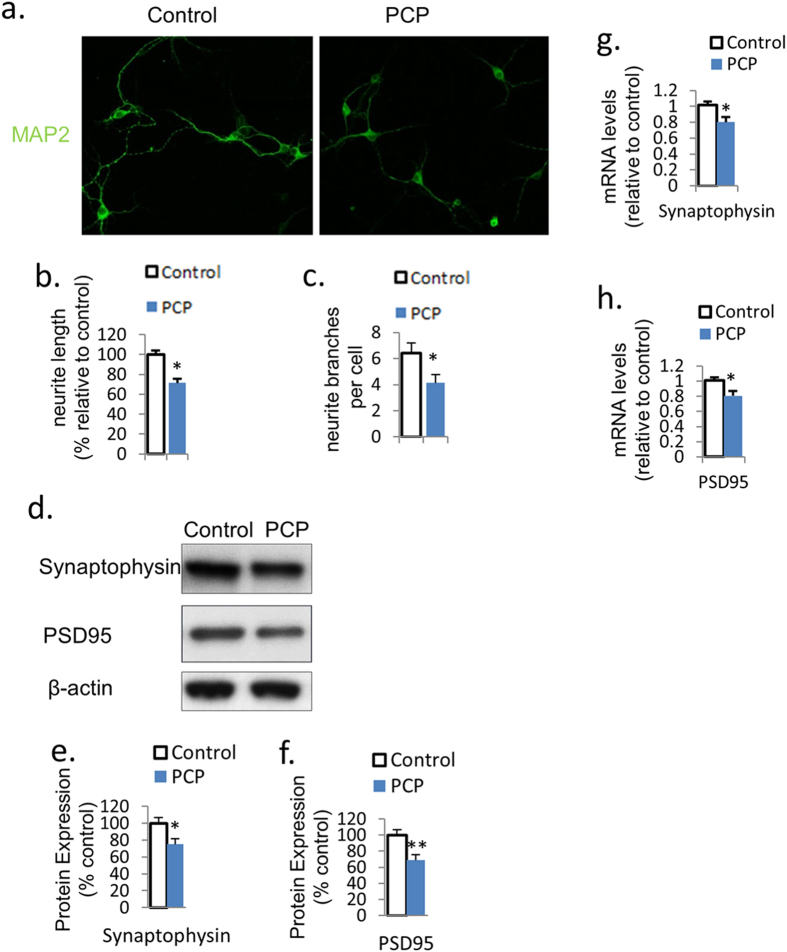
PCP suppresses neurite growth and synaptic protein expression. (**a**–**c**) Immunofluorescence with MAP2 revealed that PCP treatment reduces neurite length and the number of neurite branches. (**d**–**f**) Protein expression of synaptophysin and PSD95 was downregulated by PCP treatment in PFC neurons on DIV7. (**g**,**h**) The mRNA expression of synaptophysin and PSD95 was reduced by PCP treatment in primary PFC neurons on DIV7. Error bars indicate SEM. *p < 0.05 vs control; **p < 0.01 vs control. n = 6/group.

To examine the effect of PCP on neurite outgrowth and synaptic protein expression, we quantified neurite length and the number of branches (as markers for neurite outgrowth), and the expression of synaptophysin (pre-synaptic protein) and PDS-95 (post-synaptic protein) in primary PFC cultures treated with PCP or vehicle control for 24 h at DIV7. Immunofluorescence with MAP2 antibody revealed that PCP treatment significantly reduced both dendrite length (28.5%, *p* < 0.05) and the number of dendrite branches (35.6%, *p* < 0.05) in primary PFC cultures at DIV7 (Fig. 1a–c). Western blotting revealed that the protein expression of synaptophysin and PSD95 were both downregulated by PCP treatment for 24.9% (*p* < 0.05) and 31.5% (*p* < 0.01), respectively (Fig. 1d–f). Consistently, qRT-PCR revealed that the mRNA levels of synaptophysin and PSD95 were also reduced by PCP by 23.2% and 21.4% (both p < 0.05), respectively (Fig. 1g,h).

### PCP suppresses Akt-GSK signaling

**Figure 2:**
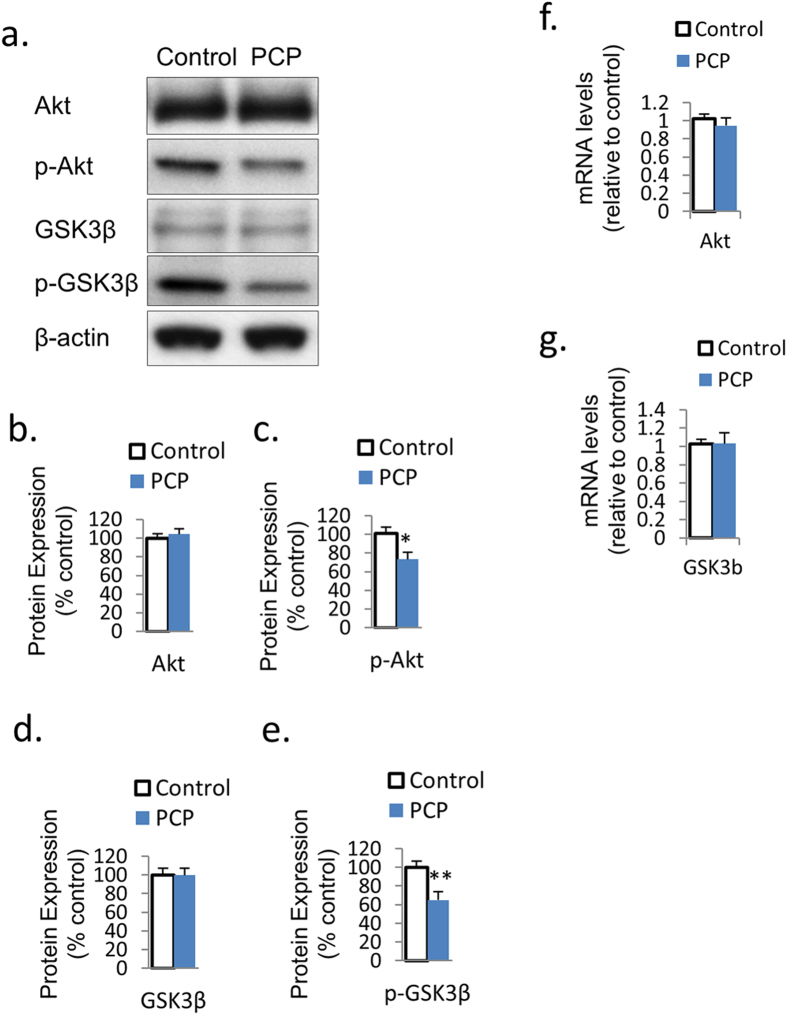
PCP suppresses Akt-GSK signaling. (**a**–**e**) Protein expression of p-Akt and p-GSK3β was reduced by PCP treatment in PFC primary cultures on DIV7, while total Akt and total GSK3β expression was unchanged. (**f**,**g**) The mRNA expression of Akt and GSK3β was unchanged under PCP treatment. Error bars indicate SEM. *p < 0.05 vs control; **p < 0.01 vs control. n = 6/group.

Akt-GSK signaling has been reported to be an important intracellular signal transduction pathway for NMDAR and NRG1’s effects on neurogenesis and synaptic plasticity^24^. To determine whether the Akt-GSK signaling cascade is affected by PCP *in vitro*, we examined the mRNA expressions of TrkB and GSK3β as well as the protein expressions of Akt, p-Akt, GSK3β, and p-GSK3β in primary PFC cultures under PCP treatment. The phosphorylated Akt (p-Akt) and GSK3β (p-GSK3β) protein expressions were both downregulated by PCP treatment compared to the control (−26.7%, *p* < 0.05; and −35.2%, *p* < 0.01, respectively), although PCP had no effect on total Akt or GSK3β expression (Fig. 2a,c,e). However, there was no difference between PCP and the control in the protein expression of total Akt and GSK3β (Fig. 2a,b,d), nor was mRNA expression of Akt or GSK3β (Fig. 2f,g).

### Olanzapine prevents PCP-induced reduction in neurite outgrowth and synaptic protein expression

**Figure 3:**
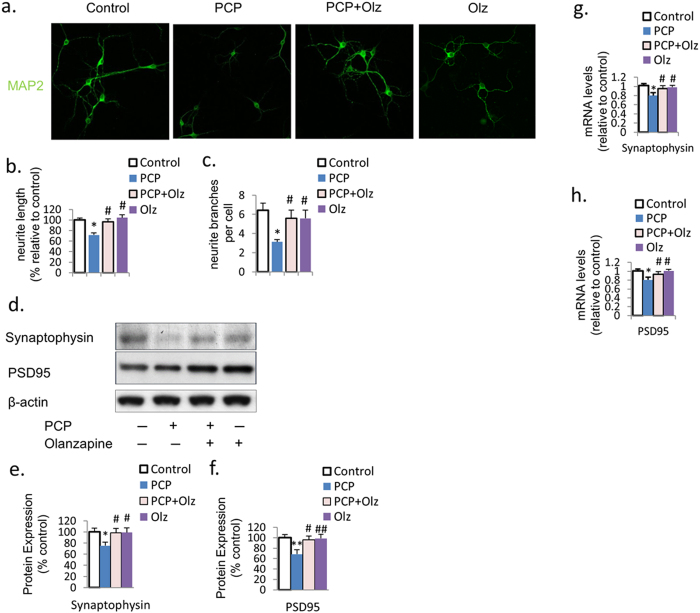
Olanzapine inhibits the PCP-induced reduction in neurite outgrowth and synaptic protein expression. (**a**–**c**) Immunofluorescence with MAP2 revealed that olanzapine co-treatment prevented the PCP-induced reduction in neurite length and number of neurite branches. (**d**–**f**) Olanzapine co-treatment also inhibited the PCP-induced reduction in protein expression of synaptophysin and PSD95. (**g**,**h**) The PCP-induced reduction in mRNA expression of synaptophysin and PSD95 was inhibited by olanzapine co-treatment. Error bars indicate SEM. *p < 0.05 vs control; **p < 0.01 vs control; ^#^*p* < 0.05 vs PCP; ^##^*p* < 0.01 vs PCP. n = 6/group.

We investigated whether the co-treatment of olanzapine with PCP could prevent the reduction in neurite outgrowth and synaptic protein expression. Immunofluorescence with MAP2 revealed that olanzapine co-treatment inhibited the PCP-induced reduction in neurite length and the number of branches (Fig. 3a–c), while the olanzapine only group didn’t have significant difference compared to control (Fig. 3a–c). Western blotting revealed that while olanzapine only didn’t have an elevation effect compared to control, olanzapine co-treatment prevented the PCP-induced reduction in the protein expression of synaptophysin and PSD95 (Fig. 3d–f). Similarly, qRT-PCR revealed that olanzapine co-treatment also prevented the decrease in the mRNA levels of synaptophysin and PSD95 (Fig. 3g,h). These results indicate that olanzapine can prevent the PCP-induced reduction in neurite outgrowth and synaptic plasticity in primary PFC cultures.

### Olanzapine reverses the PCP-induced downregulation of Akt-GSK signaling

**Figure 4:**
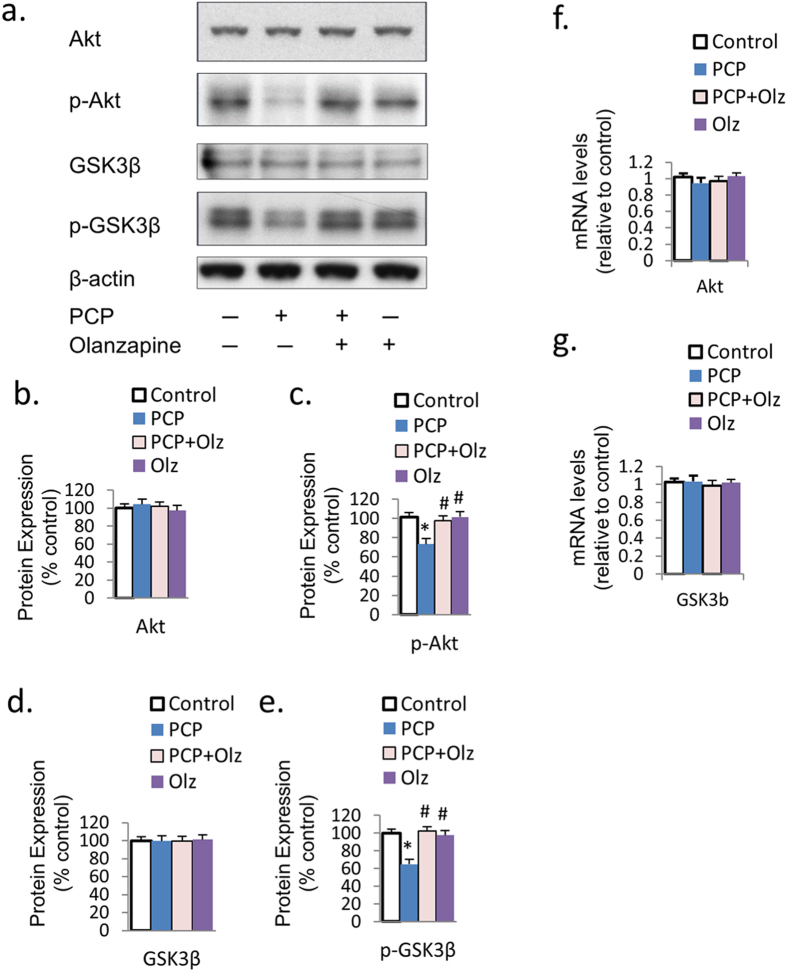
Olanzapine inhibits the PCP-induced suppression in Akt-GSK signaling. (**a**–**e**) Olanzapine co-treatment eliminated PCP-induced reduction in the protein expression of p-Akt and p-GSK3β in PFC primary cultures on DIV7. (**f**,**g**) The mRNA expression of Akt and GSK3β was unchanged under both PCP treatment and olanzapine co-treatment. Error bars indicate SEM. *p < 0.05 vs control; ^#^*p* < 0.05 vs PCP. n = 6/group.

To investigate whether olanzapine co-treatment can reverse the PCP-induced down-regulation ofn Akt-GSK signaling *in vitro,* we examined the mRNA expressions of GSK3β and Akt, and the protein expressions of Akt, p-Akt, GSK3β, and p-GSK3β in primary PFC cultures under PCP treatment or Olanzapine-PCP co-treatment. As expected, olanzapine reversed the downregulation effect of PCP on phosphorylated Akt (p-Akt) and GSK3β (p-GSK3β) protein expressions (Fig. 4a,c,e). However, the total Akt and GSK3β protein levels were not affected (Fig. 4a,b,d). Olanzapine did not affect the level of mRNA expression of Akt and GSK3β (Fig. 4f,g). These results suggest that the Akt-GSK cascade (particularly p-Akt and p-GSK3β expression), are downregulated by PCP treatment *in vitro,* but can be reversed by olanzapine.

### Olanzapine does not change neurite outgrowth and synaptic plasticity in neurons from NRG1 knockout mice

**Figure 5:**
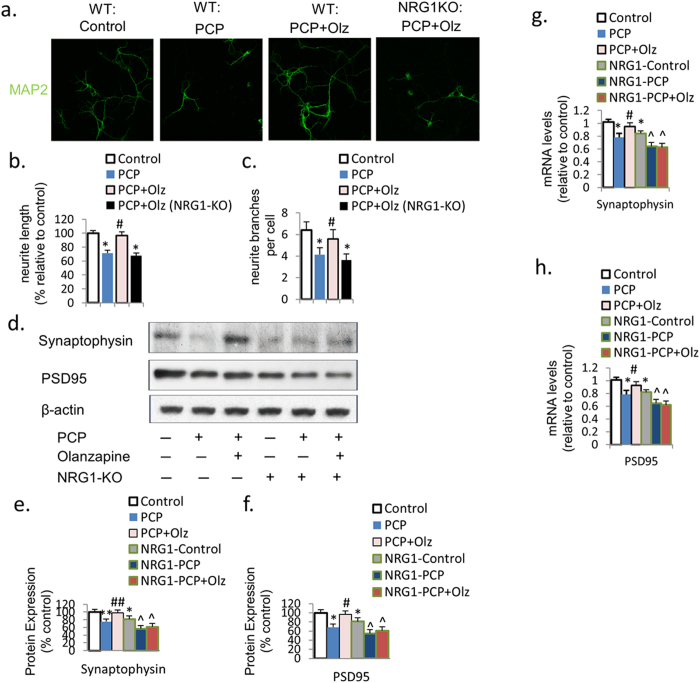
Olanzapine’s prevention effects on PCP-induced reduction in neurite outgrowth and synaptic protein expression were eliminated in neurons from NRG1-knockout mice. (**a**–**c**) The prevention effect of olanzapine on PCP-induced reduction in neurite length and neurite branches was blocked in neurons from NRG1-knockout mice. (**d**–**f**) The prevention effect of olanzapine on PCP-induced reduction in synaptophysin and PSD95 protein expression was blocked in neurons from NRG1-knockout mice. (**f**,**g**) The prevention effect of olanzapine on PCP-induced reduction in mRNA expression of synaptophysin and PSD95 was blocked in neurons from NRG1-knockout mice. Error bars indicate SEM. *p < 0.05 vs control; **p < 0.01 vs control; ^#^*p* < 0.05 vs PCP; ^##^*p* < 0.01 vs PCP; ^*p* < 0.05 vs NRG1-control. n = 6/group.

To examine whether NRG1 mediates olanzapine’s co-treatment effect on the PCP-induced reduction in neurite outgrowth and synaptic plasticity during neurodevelopment *in vitro,* we examined neurite growth and synaptic marker expression in primary PFC cultures taken from NRG1-KO mice at postnatal day 0 (PN0), and treated with PCP or olanzapine-PCP co-treatment for 24 h at DIV7. Immunofluorescence with MAP2 showed that the Olanzapine-PCP co-treatment primary PFC cultures from NRG1-KO mice had reduced dendrite length and number of dendrite branches compared to those from wild-type mice (Fig. 5a–c). Moreover, western blotting showed a significant reduction in PSD95 and synaptophysin expression in the primary PFC cultures from the NRG1-KO mice compared to their wild-type counterparts (Fig. 5d–f). Olanzapine had no effect on PCP-induced reduction in the protein expression of synaptophysin or PSD95 in neuronal cultures from NRG1-KO mice (Fig. 5d–f). Consistently, qRT-PCR revealed that olanzapine had no effect on PCP-induced reduction in the mRNA expression of PSD95 and synaptophysin in PFC neuronal cultures from NRG1-KO mice (Fig. 5g,h). These results indicate that NRG1 signaling is important in mediating olanzapine’s effect in attenuating the PCP-induced reduction in neurite outgrowth and synaptic plasticity.

### Olanzapine does not change PCP-induced downregulation of Akt-GSK signaling in primary PFC cultures from NRG1 knockout mice

**Figure 6:**
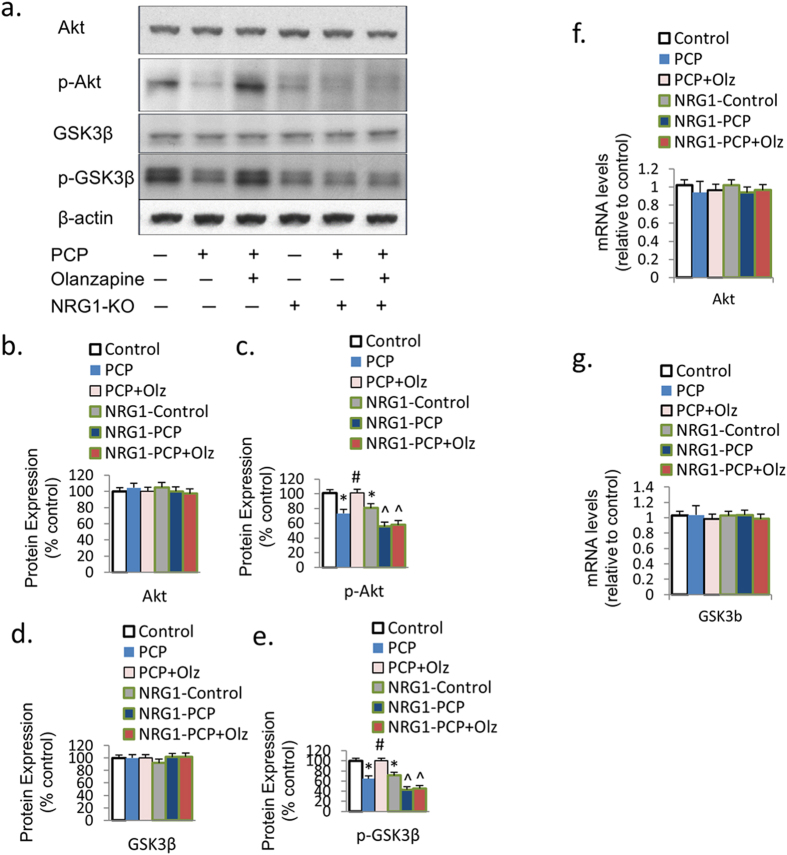
Olanzapine’s prevention effects on PCP-induced downregulation in Akt-GSK3 signaling were eliminated in neurons from NRG1-knockout mice. (**a**–**e**) The prevention effect of olanzapine on PCP-induced reduction in p-Akt and p-GSK3β expression was blocked in neurons from NRG1-knockout mice. (**f**,**g**) The mRNA expression of Akt and GSK3β was unchanged. Error bars indicate SEM. *p < 0.05 vs control; ^#^*p* < 0.05 vs PCP; ^*p* < 0.05 vs NRG1-control. n = 6/group.

**Figure 7:**
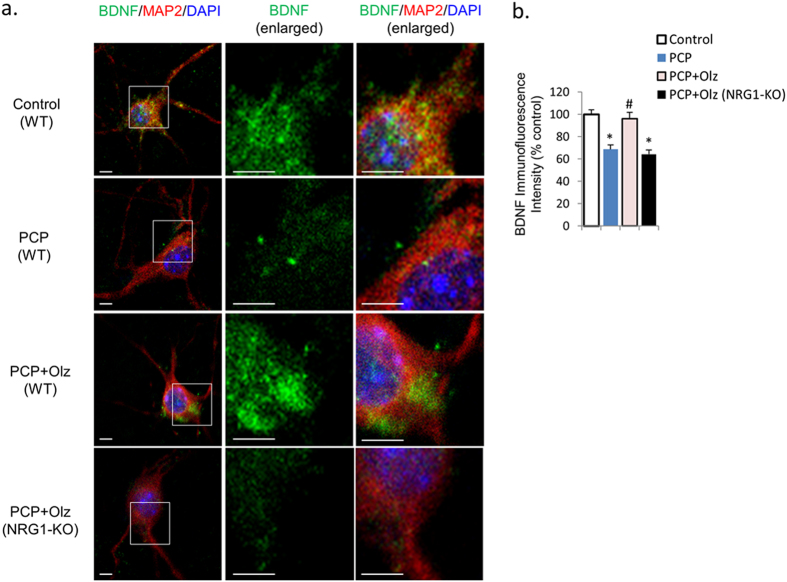
The prevention effect of olanzapine on PCP-induced reduction in BDNF immunofluorescence intensity was eliminated in neurons from NRG1-knockout mice. Error bars indicate SEM. *p < 0.05 vs control; ^#^*p* < 0.05 vs PCP. Scale bar: 5 μm. n = 6/group.

Since olanzapine co-treatment reversed PCP’s effects on Akt-GSK signaling, we hypothesized that these effects may also be blocked in PFC cultures from NRG1-KO mice during neurodevelopment. The protein expression of p-Akt and p-GSK was reduced in primary PFC cultures from NRG1-KO mice compared to those from their wild-type littermates (Fig. 6a,c,e). However, in neurons from NRG1-knockout mice, the prevention effect of olanzapine on the PCP-induced reduction in p-Akt and p-GSK3 expression disappeared (Fig. 6a,c,e), indicating that NRG1 is a critical mediator for the effect of olanzapine on preventing PCP-induced reduction in Akt-GSK signaling. Consistent with our previous findings, total Akt and GSK3β protein expression (Fig. 6a,b,d), and mRNA expression of Akt and GSK3β (Fig. 6f,g) was unchanged. Interestingly, immunofluorescence revealed that BDNF expression in NRG-KO mice primary PFC cultures compared to wild-type was reduced by 31% (Fig. 7). Finally, the levels of p-Akt, p-GSK, and BDNF expressions were highly correlated with the PSD95 expressions across all treatment groups.

**Figure 8:**
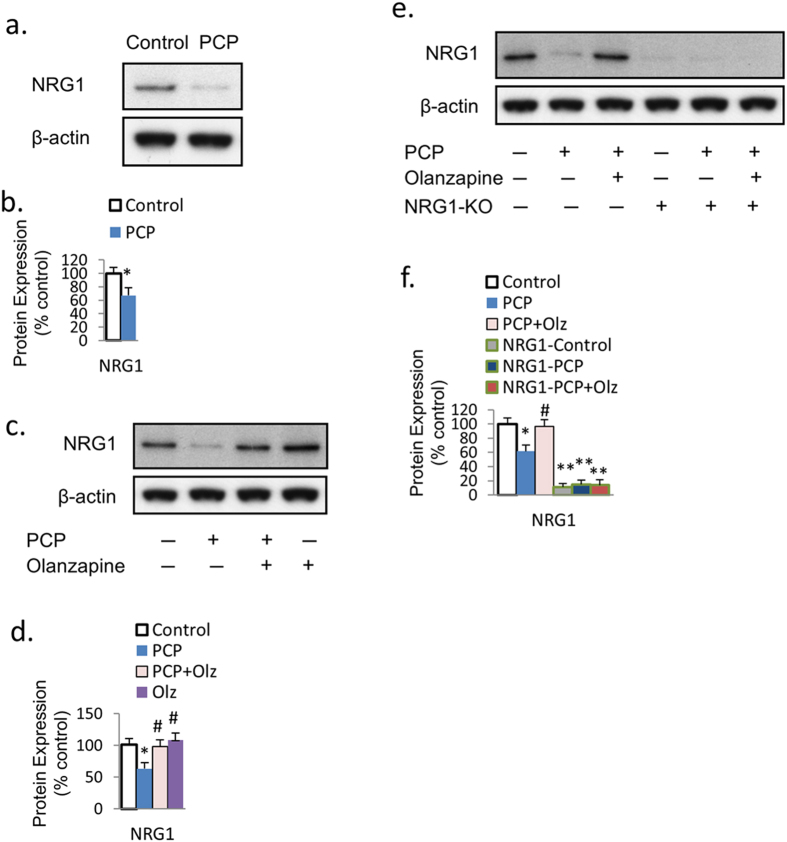
Effects of PCP and olanzapine on endogenous NRG1 expression. (**a**,**b**) PCP inhibits endogenous NRG1 protein expression. (**c**,**d**) Olanzapine reversed the inhibition effect of PCP on NRG1 protein expression. (**e**,**f**) No additional effect of PCP or olanzapine on NRG1 protein expression in neurons from NRG1-KO mice was evidenced in these neurons. Error bars indicate SEM. *p < 0.05 vs control; **p < 0.01 vs control; ^#^*p* < 0.05 vs PCP. n = 6/group.

We further investigated the effect of PCP and olanzapine on the protein expression of endogenous NRG1. PCP treatment inhibited the NRG1 expression in primary PFC neurons (Fig. 8a,b), while olanzapine reversed this effect (Fig. 8c,d). In addition, the protein expression of NRG1 in NRG1-knockout mice was significantly lower than wild type mice neuronal cultures. No further reduction effect from PCP was evidenced (Fig. 8e,f). These results suggest that PCP and olanzapine may directly affect endogenous NRG1, leading to subsequent changes in downstream markers.

## Discussion

In the present study, we found that 24 h PCP treatment suppressed neurite outgrowth and the expression of synaptic proteins (PSD95 and synaptophysin) in PFC neuronal cultures. Moreover, PCP treatment also suppressed the Akt-GSK3 signaling pathway (downregulating the phosphorylation of Akt and GSK3β), which may have led to the observed reduction in synaptic protein expression. These results are consistent with a previous report showing that PCP treatment decreases synaptic connectivity and synaptic protein expression in cultured cortical neurons, possibly via inhibiting BDNF secretion^25^. In addition, the role of Akt-GSK3 signaling in PCP-induced neurodegeneration has been demonstrated in primary neuronal cultures from the embryonic forebrains of Sprague-Dawley rats^24^. In the present study, PCP treatment decreased BDNF levels. In rodent cortical neurons, BDNF can activate phosphorylation of Akt, which could be inhibited by K252a (a TrkB inhibitor)^26^. It is possible that PCP treatment could inhibit BDNF secretion from PFC neurons, leading to the reduction of p-Akt and p-GSK3β expression. The suppressed Akt-GSK signaling may contribute to the suppression of neurite outgrowth and expression of synaptic proteins.

Olanzapine’s disinhibition effect on PCP-induced suppression in synaptic plasticity, pre-pulse inhibition, and cortical NMDAR expression has been evidenced in rodents^19^,^20^. Additionally, olanzapine could also improve dendritic outgrowth and PSD95 and BDNF expression in hippocampal neuronal cultures^18^. Therefore, in the present study, we examined the ability of olanzapine to prevent the PCP-induced suppression in neurite outgrowth and synaptic protein expression. We found for the first time at in primary PFC neurons during early developmental stage (DIV7), olanzapine can prevent PCP-induced reduction in neurite length and number of neurite branches. In addition PCP-induced downregulation in protein expressions of the presynaptic marker (synaptophysin) and the postsynaptic marker (PSD95) was also prevented by olanzapine treatment. Furthermore, olanzapine treatment also prevented the PCP-induced downregulation in the Akt-GSK3 signaling of the PFC neurons.

**Figure 9:**
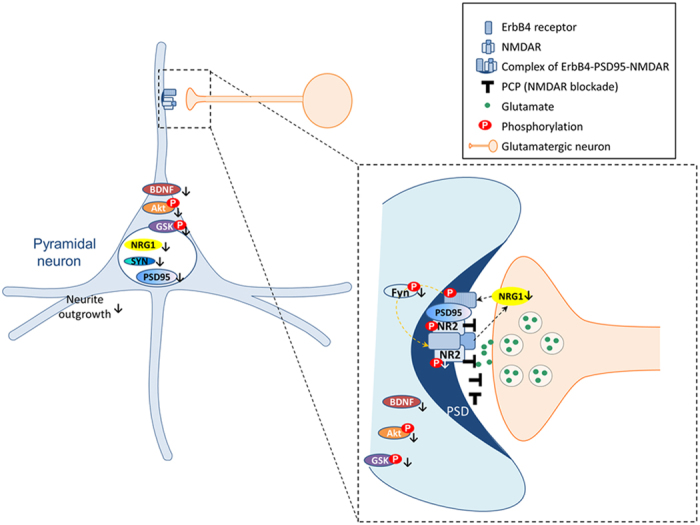
Proposed mechanism of PCP’s effect on NMDA-NRG1 interaction, synaptic protein production and neurite outgrowth. At the presence of PCP, NMDAR is blocked, which leads to an observed reduction of NRG1 expression. This reduction in NRG1 expression could be a positive feed-back effect from NMDAR to presynaptic NRG1 (an unknown mechanism), or could be a reduction in NRG1 production from the nucleus of the post-synaptic pyramidal neurons (further studies are required to confirm this). The reduction of NRG1 then leads to reduction of NRG1-ErbB4 signalling, leading to a further reduction of NMDAR function, possibly via the Fyn pathway. The hypofunction of NMDAR and reduced NRG1 could then leads to reduced BDNF expression, and down-regulation of the downstream Akt-GSK phosphorylations. This finally leads to a reduction in synaptic protein productions , including synaptophysin and PSD95, also contributing to the reduction of neurite outgrowth as observed in the present study. Fyn: Proto-oncogene tyrosine-protein kinase; NR2: NMDA receptor subunit 2; SYN: synaptophysin.

The interaction between NMDAR and NRG1 is reciprocal. It has been suggested that NRG1-ErbB4 may act downstream of NMDAR stimulation^23^. However, in the study by Bjarnadottir *et al.* (2007)^27^, NRG1 signalling increases NR2B Y1472 phosphorylation and NMDAR function, while NRG1 (∆TM)+/− mice had reduced NR2B Y1472 phosphorylation, suggesting NRG1/ErbB4 signalling may be upstream of NMDAR. Therefore, in the present study, we examined whether olanzapine can prevent the PCP-induced suppression in neurite outgrowth and synaptic protein expression in primary PFC neuronal cultures from NRG1 knockout mice compared to wild-type mice. We found that in prefrontal cortical cultures of NRG1 knockout mice, olanzapine’s treatment effect on preventing PCP-induced reduction in neurite outgrowth and synaptic protein expression was eliminated. Consistently, the effect of olanzapine on preventing PCP-induced downregulation on Akt-GSK signaling pathway and the BDNF immunofluorescence expression was also diminished in PFC cultures from NRG1-KO mice. These results suggest that the intact NRG1 pathway is critical to olanzapine’s prevention effect on PCP-induced suppression in neurite outgrowth and synaptic plasticity. In addition, the reduced NRG1 induced by PCP may also contribute to a reduced NMDAR activity (possibly through the Fyn pathway)^27^, which in combine with the blocking effect of PCP on NMDAR, leading to a further down-regulation of the downstream markers such as pAkt and pGSK (Fig. 9). We have examined the effect of olanzapine on NR2B Y1472 phosphorylation and Fyn Y420 phosphorylation with or without the Fyn autophosphorylation inhibitor PP2. We found that at the absence of PP2, olanzapine was able to up-regulate NR2B Y1472 phosphorylation as well as Fyn Y420 phosphorylation (See Supplementary Fig. S1 online). However, these effects were completely blocked by the Fyn autophosphorylation inhibitor PP2, indicating Fyn pathway is mediating the effect of olanzapine on upregulating NMDAR signaling, at least in the primary neuronal culture model of the present study.

Recently, studies have reported that the interaction between NRG1 and NMDAR^28^ and the activation of NMDAR facilitates neuronal development^29^. It is possible that the prevention effect of olanzapine on PCP-induced suppression in the Akt-GSK3 signaling pathway and neurite outgrowth was mediated through the NRG1-ErbB4 pathway. In fact, the action of NRG1-ErbB4 has been suggested to be downstream of NMDAR stimulation^23^. Therefore, eliminating NRG1 from the neuronal cultures developed from NRG1-KO mice may block olanzapine’s effect on the downstream Akt-GSK3 signaling and neurite outgrowth and synaptic protein expressions in non-mature neurons (DIV7), which relates to neuronal development. We further investigated the effect of PCP and olanzapine on the protein expression of endogenous NRG1, and found that PCP suppressed NRG1 protein expression, while olanzapine co-treatment reversed this effect. It is likely that PCP and olanzapine may act directly on endogenous NRG1 expression, leading to downstream changes in the Akt-GSK signaling pathway and neurite outgrowth and synaptic protein expressions.

According to the study by Bjarnadottir *et al.*^27^, clozapine can reverse the reduction of NR2B Y1472 phosphorylation induced by defects of NRG1 or ErbB4. However, in our study, olanzapine’s effect on correcting the PCP-induced downregulation of Akt-GSK signaling and neurite growth was eliminated by NRG1 knockout, indicating olanzapine’s effect is dependent on NRG1, while clozapine may correct the defected effect induced by NRG1 knockout, at least to some extent. Therefore, we suspect that olanzapine and clozapine may work on different mechanisms on the NRG1 and NMDAR systems, which coincide with the differences in the pharmacological profiles. Future studies are required to confirm this hypothesis.

In conclusion, our results suggest that PCP (as an NMDAR antagonist) can suppress Akt (decreasing p-Akt) and activate GSK3β (decreasing p-GSK3β), which may lead to the reduced expression of synaptic proteins (PSD95 and synaptophysin) and reduced neurite outgrowth in primary PFC neuronal cultures of wild-type mice. Olanzapine co-treatment can prevent these changes, possibly due to an elevated BDNF expression, hence normalizing Akt-GSK3 signaling, synaptic protein expression, and neurite outgrowth. Finally, this normalization effect was diminished in PFC cultures collected from NRG1 knockout mice. This suggests that NRG1 signaling is at least partly responsible for olanzapine’s treatment effect on PCP-induced suppression of neurite outgrowth and synaptic protein expression. This is the first report to examine the effect of olanzapine in a combined model of schizophrenia (NRG1 knockout and PCP). Considering the clinical relevance of PCP-induced schizophrenia-like behaviors in humans and rodents^10^,^11^, our study may provide insights into the mechanism underlining olanzapine’s effect on neurite outgrowth and synaptic protein expressions. While cautious should always be taken when extrapolating significance from primary cultures to *in vivo* or clinical studies, these results do provide interesting mechanistic insights for further studies.

## Methods

### Primary Cell Cultures

Experiments were run in 6 parallels and repeated for 3 times. Primary cortical cultures from brain tissues of postnatal day 0 (PN0) wild-type (WT) and NRG1-knockout (KO) C57BL/6 mice were prepared as described previously^30^. All experimental procedures were approved by the Animal Ethics Committee, University of Wollongong, Australia, and complied with the Australian Code of Practice for the Care and Use of Animals for Scientific Purposes^31^. Briefly, dissociated cortical cells were plated at a final density of 5 × 10^5^ cells/cm^2^, onto PDL-coated 24-well plates, containing neurobasal media (NBM) supplemented with B27. 5-FDU were added into the conditional medium (which was used to replace the NBM) to halt the growth of non-neuronal cells. Cultures were maintained at 37 ⁰C in a humidified CO_2_ incubator and used for experiments at 7 days *in vitro* (DIV7). Cells were treated with PCP at a final concentration of 1 μM, and olanzapine at a final concentration of 100 μM for 24 hours. The concentration of PCP and duration of treatment was chosen based on a previous studies showing that 3 to 48 hours of PCP treatment at the concentration of 1 μM can suppress intracellular signaling and synaptic protein expression^25^, while olanzapine at the dosage of 100 μM may improve neurite length and branches^18^.

### Western blot

After the PCP or olanzapine treatments, cells were harvested with lysis buffer (containing NP40, Protease Inhibitor Cocktail, 1 mM PMSF and 0.5 mM β-glycerophosphate). Total protein concentrations were determined by DC-Assay (Bio-Rad, Hercules, CA), and detected using a SpectraMax Plus384 absorbance microplate reader (Molecular Devices, Sunnyvale, CA). Samples were heat-treated in Laemmli buffer at 95 °C, loaded to 8% SDS-PAGE gels for fractionation, and then transferred onto Immun-BlotTM PVDF membranes (Bio-Rad, Hercules, CA). The block consists of 5% BSA in TBST. The membranes were then incubated with synaptophysin, PSD95 (Life Technologies, NSW; dilution factor 1:1000), BDNF, NRG1 (Santa Cruz Biotechnology, Santa Cruz, CA; dilution factor 1:500), Akt, p-Akt, GSK3β, and p-GSK3β (Cell Signalling Technology, Beverly, MA; dilution factor 1:1000) antibodies in TBST containing 1% BSA overnight at 4 °C. Secondary antibodies were anti-rabbit IgG conjugated with horseradish peroxidase (Santa Cruz Biotechnology, Santa Cruz, CA; dilution factor 1:5000). For visualization, ECL detection reagents were used and films were exposed on the AGFA CP1000 Tabletop Processor (AGFA Healthcarem Scoresby, VIC). Films were analysed using Quantity One software connected to a GS-690 Imaging Densitometer (Bio-Rad, Hercules, CA).

### Quantitative real-time PCR (qRT-PCR)

The qRT-PCR protocol was adopted from our previous work^32^,^33^. Briefly, total RNA was extracted using the PureLink RNA extraction kit (Life Technologies, NSW) according to the manufacturer’s protocol. First-strand cDNA was synthesized with the VILO cDNA synthesis kit (Life Technologies, NSW) with 20 μL reaction volume. qRT-PCR was carried out in triplicates using TaqMan Gene Expression Assays (Life Technologies, NSW) on LightCycler480+ (Roche, Penzberg, Germany). The endogenous control genes were selected using the geNorm^TM^ Reference Gene SelectionKit (Primerdesign, Southampton, UK). The results were normalized to mouse β-actin and GAPDH (cat. no. 4331182; Life Technologies, NSW), and were expressed as folds different from control. The assay identification of the target gene were Mm00436850_m1 (synaptophysin), Mm00492193_m1 (PSD95), Mm01331626_m1 (Akt), and Mm00444911_m1 (GSK3b) (Life Technologies, NSW). The qPCR data was analysed using the “delta-delta Ct method”^34^ for comparing relative expression results between treatments. Experiments were performed in triplicate.

### Immunofluorescence microscopy

Cells were grown to approximately 70% confluence on glass coverslips and treated with PCP, PCP + Olanzapine, or vehicle for 24 h before being fixed in 4% paraformaldehyde. Cells were then washed in PBS, and permeabilized with 0.3% Triton X-100 in PBS for 10 min. After blocking the coverslips with 5% normal goat serum for 1 h at room temperature, primary antibody incubations were performed in 1% goat serum in PBS overnight at 4 °C, followed by incubation in a secondary antibody cocktail of Alexa Fluor 488, 594, and DAPI (Life Technologies, NSW) for 2 h at room temperature. Cells were viewed using a 40× or a 63× oil immersion objective on a DMI6500B confocal microscope (Leica, Mannheim, Germany).

### Statistics

SPSS (version 15; Chicago, IL) was used for statistical analysis. One-way analysis of variance (ANOVA) or two-way ANOVAs with post-hoc Tukey’s tests were performed for multiple comparisons. Data were expressed as mean ± SEM, and *p* < 0.05 was considered statistically significant.

## Additional Information

**How to cite this article**: Zhang, Q. *et al.* Olanzapine Prevents the PCP-induced Reduction in the Neurite Outgrowth of Prefrontal Cortical Neurons via NRG1. *Sci. Rep.* **6**, 19581; doi: 10.1038/srep19581 (2016).

## Supplementary Material

Supplementary Information
