# Supplementary material for: Olanzapine Prevents the PCP-induced Reduction in the Neurite Outgrowth of Prefrontal Cortical Neurons via NRG1
Source: Sci Rep. 2016 Jan 19;6:19581. doi: 10.1038/srep19581 (PMC4726088; doi:10.1038/srep19581)
Supplement: Supplementary Information [file srep19581-s1.pdf]

Olanzapine Prevents the PCP-induced Reduction in the Neurite Outgrowth of Prefrontal Cortical Neurons via NRG1

Qingsheng Zhang [a, b], Yinghua Yu [a, b, c] and Xu-Feng Huang* [a, b, c]

Supplementary Figure S1

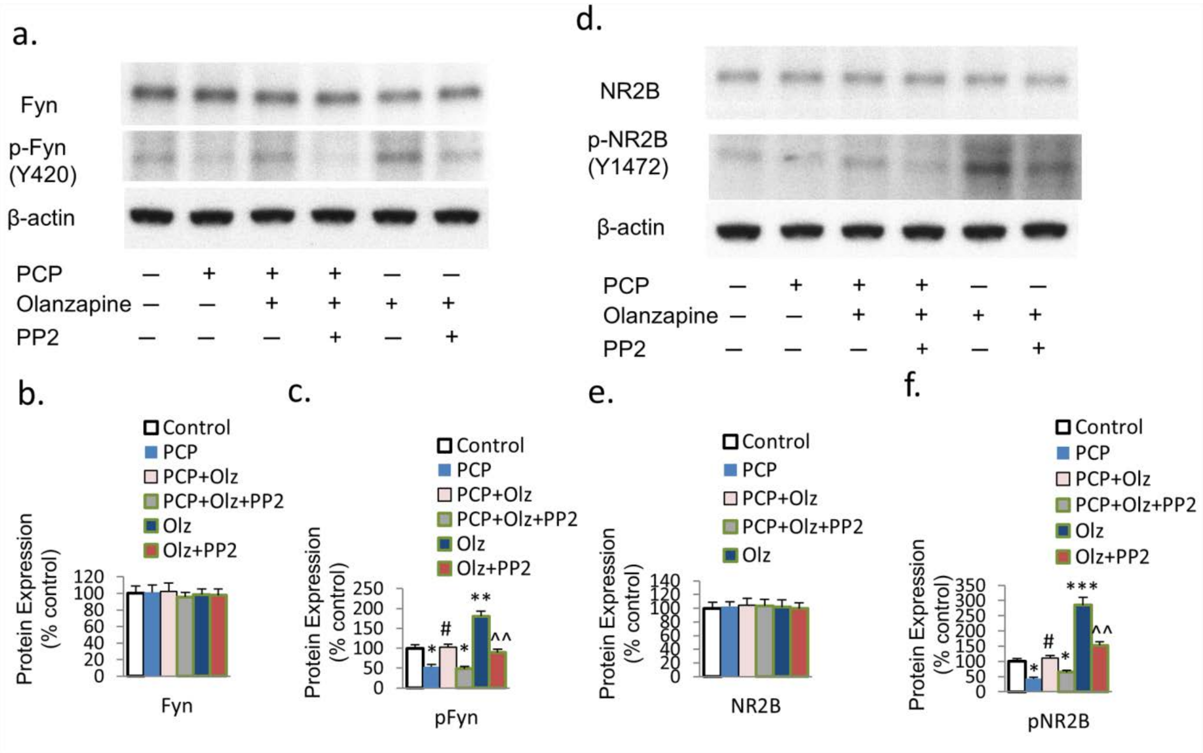

Supplementary Fig. 1 Olanzapine's effects on pFyn and pNR2B were via PP2. (a-c) Olanzapine prevented PCP's reduction effect on Fyn phosphorylation at tyrosine 420, which was blocked by PP2. Olanzapine's elevation effect on Fyn phosphorylation (Y420) was also blocked by PP2. (d-f) Olanzapine prevented PCP's reduction effect on NR2B phosphorylation at tyrosine 1472, which was blocked by PP2. Olanzapine's elevation effect on NR2B phosphorylation (Y1472) was also blocked by PP2. (n=6/group) * *P*<0.05 vs control; ** *P*<0.01 vs control; *** *P*<0.001 vs control; #*P*<0.05 vs PCP; ^^ *P*<0.01vs Olz.
